# Effects of the Bone/Bone Marrow Microenvironments on Prostate Cancer Cells and CD59 Expression

**DOI:** 10.1155/2020/2753414

**Published:** 2020-04-06

**Authors:** Bo Yan, Yan Li, Shaoju Min, Peng Zhang, Bin Xu, Zhen Wang, Wei Zhang, Jiasheng Chen, Guangheng Luo, Chunxiao Liu

**Affiliations:** ^1^Department of Urology, Guizhou Provincial People's Hospital, Medical College of Guizhou University, Guiyang, Guizhou, China; ^2^Department of Urology, Zhujiang Hospital, Southern Medical University, Guangzhou, Guangdong, China; ^3^Department of Neurosurgery, Zhujiang Hospital, Southern Medical University, Guangzhou, Guangdong, China; ^4^Department of Clinical Laboratory, The Affiliated Hospital of Guizhou Medical University, Guiyang, Guizhou, China

## Abstract

**Objective:**

To evaluate the effects of human bone marrow mesenchymal stem cells (hBMSCs) and osteoblasts (hFOB1.19) on PC3 prostate cancer cells.

**Methods:**

To simulate the *in vivo* interaction between the bone/bone marrow microenvironments and prostate cancer cells, we established cocultures of PC3 cells with hBMSC or hFOB1.19 cells and evaluated their effects on the proliferation, cell cycle distribution, cell migration, and invasion of PC3 cells. Quantitative reverse transcription polymerase chain reaction was used to detect *CD59* mRNA expression in PC3 cells. The expression of receptor activator of nuclear factor- (NF-) *κ*B (RANK), RANK ligand (RANKL), osteoprotegerin (OPG), CD59, NF-*κ*B (p50 subunit), and cyclin D1 in PC3 cells was analyzed by immunofluorescence and western blotting.

**Results:**

hBMSCs and hFOB1.19 cells enhanced the proliferation, migration, and invasion of PC3 cells; increased the proportion of PC3 cells in the S and G_2_/M phases of the cell cycle; and upregulated RANK, RANKL, OPG, CD59, cyclin D1, and NF-*κ*B (p50 subunit) expression by PC3 cells. The RANKL inhibitor, scutellarin, inhibited these effects in PC3-hFOB1.19 cocultures.

**Conclusion:**

hBMSCs and hFOB1.19 cells modulate the phenotype of PC3 prostate cancer cells and the expression of CD59 by activating the RANK/RANKL/OPG signaling pathway.

## 1. Introduction

Prostate cancer is one of the most common malignant tumors among men in Europe and the United States and the second most common male cancer worldwide [1]. If prostate cancer metastasizes, it preferentially localizes to the bone, which is observed in approximately 65–80% of patients with advanced-stage disease [2, 3]; however, the molecular mechanisms that mediate bone metastasis in patients with prostate cancer are not well characterized. Early-stage prostate cancer is androgen-dependent and typically asymptomatic; therefore, most patients are diagnosed with advanced-stage disease. Once metastasis occurs, prostate cancer often becomes androgen-independent and progresses to hormone-refractory prostate cancer (HRPC), which is insensitive to endocrine therapy, and for which, there is no well-established standard of care [4].

Within the tumor microenvironment, interactions between innate cells and tumor cells promote changes in cytokine concentrations and extracellular matrix components proximal to the tumor. This exchange affects various tumor cell activities such as growth, invasion, metastasis, and response to therapeutic drugs. Some studies have shown that dissemination of tumor cells to the bone is not proportional to the actual incidence of metastases, indicating that the bone microenvironment greatly favors the localization and growth of metastatic prostate cancer cells [5], particularly, HRPC cells. Metastatic bone cancer tumors generally present as osteolytic lesions, whereas bone metastases of prostate cancer are primarily osteogenic lesions, suggesting that prostate cancer cells may affect the growth of osteoblasts and bone remodeling [6–8].

The receptor activator of nuclear factor- (NF-) *κ*B (RANK); its ligand (RANKL); and the antagonist, osteoprotegerin (OPG); comprise an important cytokine system within the bone microenvironment that can mediate tumor growth. RANK is expressed on the surface of osteoclast precursors and mature osteoclasts, whereas RANKL and OPG, a soluble decoy receptor for RANKL, are expressed by osteoblasts and bone marrow stromal cells. When RANKL (expressed by osteoblasts) binds to RANK on the surface of osteoclast precursor cells, it induces the differentiation of the precursors into mature osteoclasts, whereas binding of RANKL to RANK on the surface of mature osteoclasts further activates the bone resorption capacity of osteoclasts. Studies outside China have reported that RANK, RANKL, and OPG are expressed by prostate cancer tissues, suggesting that RANK, RANKL, and OPG may promote the incidence and growth of prostate cancer. Moreover, Christoph et al. demonstrated that RANK, RANKL, and OPG are expressed by primary prostate cancer cells and their expression levels significantly correlate with the Gleason score, serum prostate-specific antigen (PSA) levels, and disease progression [9]. In another study, researchers found that RANK, RANKL, and OPG were expressed more strongly by prostate cancer metastases (to the bone and lymph nodes) than by the primary tumor. Subgroup analysis demonstrated that bone metastases produced more RANK, RANKL, and OPG than lymph node metastases and that their expression levels correlated with clinical staging, the Gleason score, and the PSA level [10].

CD59 is a membrane-bound protein that inhibits the formation of the complement membrane attack complex (MAC). It protects cells from MAC-induced cell lysis, thus promoting tumor cell immune escape. In previous studies, we found higher levels of CD59 expression in bone metastases of patients with prostate cancer than in primary prostate cancer lesions, suggesting that CD59 may play an important role in the metastasis of prostate cancer to the bone.

In this study, we cocultured osteoblasts (hFOB1.19 cells) and their precursors (human bone marrow mesenchymal stem cells (hBMSCs)) with prostate cancer (PC3) cells to simulate the effects of the bone and bone marrow microenvironments on prostate cancer cells and investigated the relationship between the RANKL/RANK/OPG signaling pathway and CD59 expression.

## 2. Materials and Methods

### 2.1. Cell Lines and Cell Culture

Human bone marrow mesenchymal stem cells (hBMSCs), human osteoblasts (hFOB1.19 cells), and PC3 prostate cancer cells were purchased from the American Type Culture Collection (Manassas, VA, USA) and cultured in a complete growth medium after thawing, as follows. Human BMSCs were cultured in Dulbecco's modified Eagle's medium- (DMEM-) L (cat. no. D5546; Sigma, St. Louis, MO, USA) with 10% fetal bovine serum (FBS) (cat. no. 10100139C; Gibco, Australia), 1% (*v*/*v*) l-glutamine (cat. no. 25030149; Invitrogen, Carlsbad, CA, USA), penicillin (100 U/mL), and streptomycin (100 *μ*g/mL). Human FOB1.19 cells were cultured in DMEM-Ham's F12 (cat. no. DF-042; Millipore, USA) with 10% FBS, 1% (*v*/*v*) l-glutamine, penicillin (100 U/mL), and streptomycin (100 *μ*g/mL). PC3 cells were cultured in RPMI 1640 (cat. no. R5886; Sigma) with 10% FBS, 1% (*v*/*v*) l-glutamine, penicillin (100 U/mL), and streptomycin (100 *μ*g/mL). All cells were grown in an incubator containing 5% CO_2_ at 37°C. Culture medium was replaced as needed. PC3 was derived from bone metastasis of prostate cancer patients, which may have more biological characteristics of prostate cancer bone metastasis cells. hFOB1.19 is an osteoblast transfected with human SV40 gene. It was derived from the limb bone tissue of aborted fetus and expresses SV40 T antigen. It was used to simulate the bone microenvironment. hBMSCs, derived from normal human bone marrow, were used to simulate the bone marrow microenvironment.

Indirect contact was used for coculturing. For detection of cell proliferation by MTS assays, 96-well ordinary culture plates plus conditioned medium were used. In other experiments, transwell cell culture plates (cat. no. REF353097; BD) with 8 *μ*m pore sizes were used for cell migration and invasion experiments, and those with 3 *μ*m pore sizes were used for other experiments.

### 2.2. Conditioned Medium

When hBMSCs reached the logarithmic growth phase, the medium was changed to fresh DMEM-L with 10% FBS. After 48 h, the medium was collected, centrifuged at 400 × *g* for 10 min to remove cell debris, filtered through a 0.22 *μ*m filter to remove bacteria, and stored at -20°C.

When hFOB1.19 cells reached the logarithmic growth phase, the medium was changed to fresh DMEM-Ham's F12 with 10% FBS. After 48 h, the medium was collected, centrifuged at 400 × *g* for 10 min, filtered through a 0.22 *μ*m filter, and stored at -20°C.

### 2.3. Antibodies

The following primary antibodies were used in this study: anti-CD59 [MEM-43] (cat. no. ab9182; Abcam, Cambridge, UK), anti-cyclin D1/FSTL3 (cat. no. bs-0623R; Bioss), anti-osteopontin (cat. no. ab33046; Abcam), anti-NF-*κ*B p105/p50 [E381] (cat. no. ab32360; Abcam), anti-RANK [EPR4740(N)] (cat. no. ab182158; Abcam), anti-RANKL [12A668] (cat. no. ab45039.7; Abcam), and anti-RANK (cat. no. ab222215; Abcam).

The following secondary antibodies were used in this study: goat anti-mouse IgG H&L (Alexa Fluor 488; cat. no. ab150113; Abcam), goat anti-rabbit IgG H&L (Alexa Fluor 488; cat. no. ab150077; Abcam), goat anti-rabbit IgG H&L (horseradish peroxidase (HRP)) preadsorbed (cat. no. ab97080; Abcam), and goat anti-mouse IgG H&L (HRP) preadsorbed (cat. no. ab97040; Abcam).

### 2.4. Cell Proliferation by MTS Assay

PC3 cells in the logarithmic growth phase were seeded into 96-well plates at 1 × 10^4^ cells/well. After the cells adhered to the culture plates, the culture medium was changed, and the cells were divided into the following four groups: (1) PC3 control group: PC3 cells were cultured in PC3 complete culture medium; (2) PC3-hBMSC coculture group: PC3 cells were cultured in 2/3 volume PC3 complete culture medium plus 1/3 volume hBMSC-conditioned medium; (3) PC3-hFOB1.19 coculture group: PC3 cells were cultured in 2/3 volume PC3 complete culture medium plus 1/3 volume hFOB1.19-conditioned medium; and (4) PC3-hFOB1.19 coculture+scutellarin group: PC3 cells were cultured in 2/3 volume PC3 complete culture medium, 1/3 volume hFOB1.19-conditioned medium, and 100 *μ*m scutellarin (cat. no. HY-N0751; MCE). Proliferation was assessed 0, 24, 48, and 72 h after the cells were divided into different culture groups. The CellTiter 96 AQueous One Solution Cell Proliferation Assay detection reagent (cat. no. G3582; Promega, Madison, WI, USA) was added to the culture media at 10 *μ*L reagent/100 *μ*L culture medium. After 4 h, the optical density was detected at a wavelength of 490 nm using a microplate reader. The proliferation rate was calculated using the following formula: proliferation rate = (average optical density value at other time points/average optical density value at 0 h–1 h) × 100%.

### 2.5. Cell Cycle Analysis by Flow Cytometry

Cells were divided into the four experimental groups described above in Cell Proliferation by MTS Assay; however, in this assay, PC3 cells were seeded into the lower chambers of 6-well transwell culture plates, and hFOB1.19 cells or hBMSCs were seeded in the upper chambers. In the coculture groups, complete culture media for each cell type was added at a 1 : 1 volume.

Cells in the logarithmic growth phase were collected (1 × 10^6^ cells/group) and washed twice with phosphate-buffered saline (PBS). Next, ethanol was added to a final concentration of 75% and the cells were incubated overnight at 4°C. Before staining, the cell suspensions were centrifuged at 500 × *g* for 5 min, the supernatants were discarded, and the cells were washed again with PBS. Propidium iodide (PI) staining solution was reconstituted in PBS, and 500 *μ*L of the staining solution was added to the cells at the following final concentrations: 50 *μ*g/mL PI, 100 *μ*g/mL RNase A, 0.2% Triton X-100, and 1 mg/mL sodium citrate. The cells were then incubated 30 min at 4°C in the dark. Cells (2 × 10 [4]–3 × 10 [4]) were run through a flow cytometer (FACSCalibur; BD), and cell cycle distribution was analyzed using ModFit software.

### 2.6. Transwell Migration Assay

Cells were seeded and cultured as described in Cell Cycle Analysis by Flow Cytometry into 24-well transwell culture plates and incubated for 48 h at 37°C with 5% CO_2_. The chambers were then disassembled, and the cells remaining on the upper surface of the chamber divider were removed using cotton swabs. Cells attached to bottom of the chamber dividers were fixed with 4% paraformaldehyde for 20 min, washed once with PBS, stained with crystal violet for 10 min, washed again with PBS, and photographed. Cells that had passed through the pores to the underside of the divider were examined and counted with a microscope.

### 2.7. Transwell Invasion Assay

Cells were seeded and cultured as described in Transwell Migration Assay with the following differences. Matrigel (cat. no. 356234; BD Bioscience) was incubated at 4°C overnight, diluted with precooled serum-free medium at a volume ratio of 1 : 3 (Matrigel : medium), and 40 *μ*L of the Matrigel solution was added to each of the upper chambers of 24-well transwell culture plates. The plates were incubated at 37°C for 2 h to allow the Matrigel to solidify, then 100 and 600 *μ*L serum-free media were added to the upper and lower chambers, respectively, and the plates equilibrated overnight in a 37°C incubator. The rest of the experiment is the same as cell migration.

### 2.8. Quantitative Reverse Transcription Polymerase Chain Reaction (qRT-PCR)

Cells were seeded and cultured as described in Cell Cycle Analysis by Flow Cytometry. Cells from each treatment group were collected, transferred to RNase-free EP tubes, and washed with PBS three times. RNA was extracted with TRIzol (cat. no. 15596026; Invitrogen), and RNase-free DNase I (Promega) was added to remove any DNase prior to assessing the purity and integrity of the total RNA. Reverse transcription was performed using a reverse transcription kit (cat. no. A5000; Promgea), according to the manufacturer's instructions. We used 18S RNA as an internal reference gene and the following qRT-PCR primers: CD59-F1 GGCCTGTGACTTTCTAACCT and CD59-R1 TGAGAGACACAAGTCCCTCTT (140 bp), 18S-F1 CCTGGATACCGCAGCTAGGA and 18S-R1 GCGGCGCAATACGAATGCCCC (112 bp). We used a SYBR® Green detection assay (cat. no. 11733046; Invitrogen), according to the manufacturer's instructions, and ran the qRT-PCRs on an ABI PRISM 7500 (ABI) using the following conditions: predenaturation at 95°C for 5 min and 40 cycles of 95°C for 15 s and 60°C for 32 s. Each sample was tested in triplicate. Melting curve analysis was performed at 60°C–95°C. The relative expression of *CD59* mRNA was calculated using the 2^-*ΔΔ*Ct^ method.

### 2.9. Protein Expression by Immunofluorescence

Coverslips were cut into suitable sizes, soaked in concentrated sulfuric acid overnight, rinsed with tap water five times, washed in an ultrasonic cleaner with double-distilled water three times, sterilized under high pressure, and dried. The coverslips were then transferred to 6-well transwell culture plates using sterile tweezers. Cells were seeded onto the coverslips and cultured for 24–48 h. The culture medium was removed, and the adherent cells were washed with PBS. The cells were fixed in 4% paraformaldehyde for 30 min, placed in a sterile container, washed with PBS for 5 min (×3), treated with 0.2% Triton X-100 for 5 min, and again washed with PBS for 5 min (×3). The cells were then blocked with 10% normal goat serum (cat. no. ab7481; Abcam) for 30 min, incubated with primary antibodies (1 : 100) at 4°C overnight, washed with PBS for 5 min (×3), and incubated with fluorescent-labeled secondary antibodies (1 : 200) in a wet box at room temperature (20–25°C) for 1 h in the dark. After incubation, cells were washed with PBS for 5 min (×3), incubated in 4′,6-diamidino-2-phenylindole at room temperature in a wet box in the dark for 5 min, washed again with PBS for 5 min, and washed twice with water for 5 min. Fluorescence antifade reagent was used for mounting, and photographs were acquired with a fluorescence microscope (cat. no. DMI6000B; Leica).

### 2.10. Protein Expression by Western Blot Analysis

Cytoplasmic proteins and nuclear proteins were extracted using an NE-PER Nuclear and Cytoplasmic Extraction Reagent kit (cat. no. 78833; Thermo Fisher). Total protein was extracted from the cells with RIPA lysis buffer, and protein was quantified using a BCA Protein Assay Kit (cat. no. 23227; Thermo Fisher).

The lysates (30 *μ*g protein/sample) were placed in 0.5-mL centrifuge tubes, and 5x sodium dodecyl sulfate (SDS) loading buffer was added to a final concentration of 1x SDS. The samples were boiled at 100°C for 5 min to denature the protein and then loaded into each lane. Proteins were run through 5% stacking gels (80 V for 50 min) then separated at 120 V using the following gels: CD59: 15%; cyclin D1, RANKL; and OPG: 12%; and RANK and NF-*κ*B (p50): 10%. Bromophenol blue was used to monitor the progression of the proteins through the gels.

In an ice bath, the proteins were transferred to polyvinylidene difluoride membranes (cat. no. IPVH00010; Model: 0.45 *μ*m; Millipore) by electrophoresis (60 V for 120 min). To visualize the proteins, the membranes were placed on a shaker for 5 min in 1x Ponceau S solution and washed with water.

Next, the membranes were rinsed with TBST three times, rocked on a shaker in blocking solution for 1 h at room temperature, and washed with TBST for 5 min (×3). The membranes were incubated with their respective primary antibodies (diluted with TBST at a ratio of 1 : 1000) at room temperature for 1–2 h. Subsequently, the membranes were rocked on a shaker with TBST for 5 min (×3), then incubated with horseradish peroxidase-labeled secondary antibodies (1 : 5000 dilution) at 37°C for 1 h. The membranes were washed with TBST for 5 min (×3), then rinsed with double distilled water for 2 min (×3). The fluorescent substrate was dripped evenly onto the surface of the membranes, and the membranes were incubated for 5 min prior to the visualizing of the proteins using a gel imaging system (ChemiDoc XRS; Bio-Rad, Hercules, CA, USA). The densities of the grey values corresponding to the protein bands were quantified using Image J software.

### 2.11. Statistical Analysis

For each experiment, triplicate samples were analyzed for each variable. The data are expressed as the mean ± standard deviation (SD). SPSS 25 (IBM, Inc., Chicago, IL, USA) was used for data analysis. Student's *t*-tests were used for comparisons of mean values between two groups of independent samples. One-way analysis of variance was used for comparisons of mean values among multiple samples. The SNK method and Bonferroni method were used for data analysis when the variance was homogeneous. Dunnett's T3 and Dunnett's C methods were used for data analysis when the variance was not homogeneous. Differences of *P* < 0.05 were considered statistically significant.

## 3. Results

### 3.1. hBMSC and hFOB1.19 Cells Promote the Proliferation of PC3 Cells

**Figure 1:**
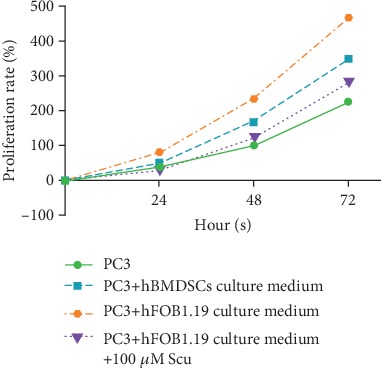
Growth of PC3 cells by MTS assay. hBMSCs: human bone marrow mesenchymal stem cells; hFOB1.19: human SV40-transfected osteoblasts; PC3: human prostate cancer cells; Scu: scutellarin. Data represent the mean ± SD of three replicates.

Using MTS cell proliferation analyses, we found that media conditioned by hBMSC and hFOB1.19 cells significantly enhanced the proliferation of PC3 cells, whereas 100 *μ*m scutellarin inhibited their proliferation (*P* < 0.01, Figure 1).

### 3.2. Cell Cycle Analysis by Flow Cytometry

**Figure 2:**
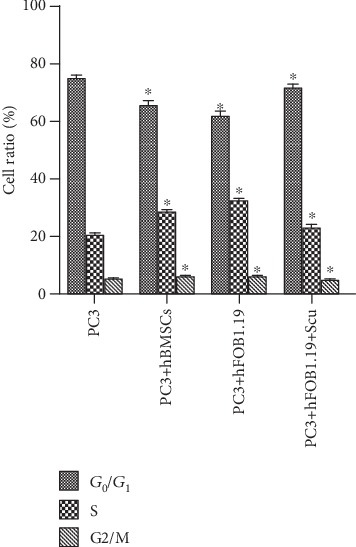
The effects of hBMSC and hFOB1.19-conditioned media on the cell cycle distribution of PC3 cells by flow cytometry. hBMSCs: human bone marrow mesenchymal stem cells; hFOB1.19: human SV40-transfected osteoblasts; PC3: human prostate cancer cells; Scu: scutellarin. Statistical analysis was performed between the same cell cycle phase of each group. ^∗^*P* < 0.05.

**Figure 3:**
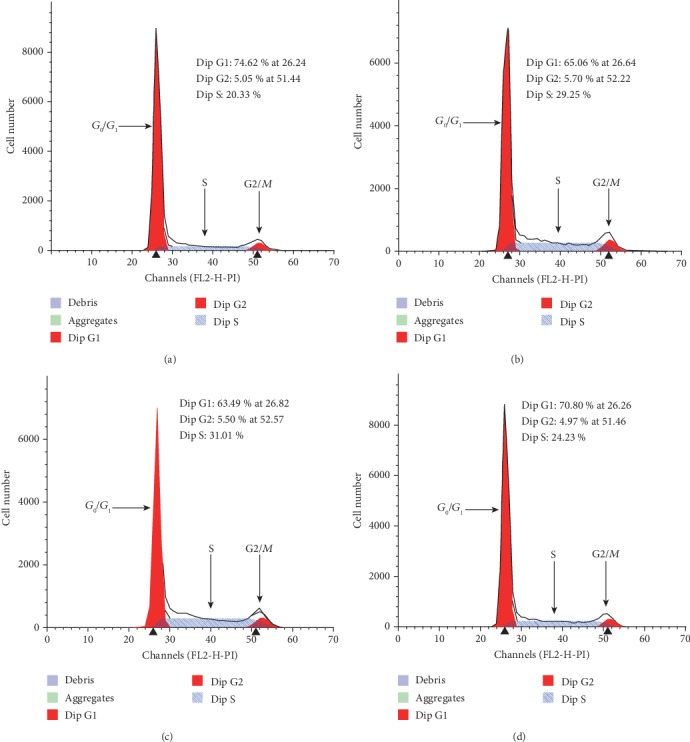
Representative images of PC3 cell cycle analysis. (a) Control group (PC3 cells), (b) PC3 cells cocultured with hBMSC cells, (c) PC3 cells cocultured with hFOB1.19 cells, and (d) PC3 cells cocultured with hFOB1.19 cells+scutellarin. The ordinate shows the number of cells counted, and the abscissa shows DNA content. G_2_/G_1_ was 2.0 (i.e., the cells were tetraploid in the G_2_ phase and diploid in the G_1_ phase, with a ratio of 2).

Cell cycle distribution by flow cytometry is shown in Table 1 and Figure 2.  Figure 3 illustrates the effects on the cell cycle distribution of PC3 cells of coculturing them with hBMSC or hFOB1.19 cells. We found that the proportion of PC3 cells cocultured with hBMSCs and hFOB1.19 cells in G_0_/G_1_ phase was lower than that of the control group (*P* < 0.05), whereas the proportion of cells in the S phase and G_2_/M phase was higher than that of the control group (*P* < 0.05). These data suggest that hBMSCs and hFOB1.19 cells promote cell cycle progression/proliferation of PC3 cells, and the RANKL inhibitor, scutellarin, inhibits these effects.

### 3.3. Migration and Invasion of PC3 Prostate Cancer Cells

**Figure 4:**
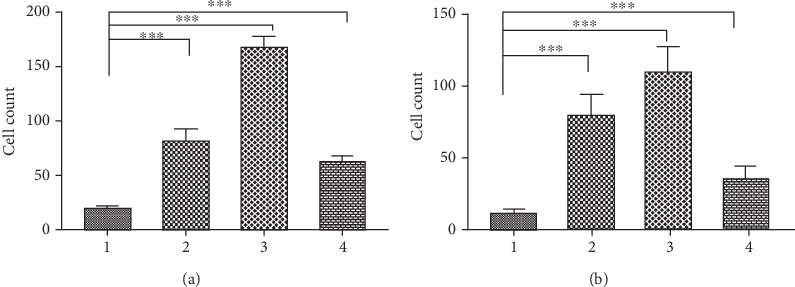
Effects of coculture on PC3 cell migration and invasion. (a) Migration experiment. (b) Invasion experiment. (1) Control group (PC3 cells); (2) PC3 cells and hBMSC coculture group; (3) PC3 cells and hFOB1.19 coculture group; (4) PC3 cells and hFOB1.19 coculture plus scutellarin (Scu) group. ^∗∗∗^*P* < 0.001.

**Figure 5:**
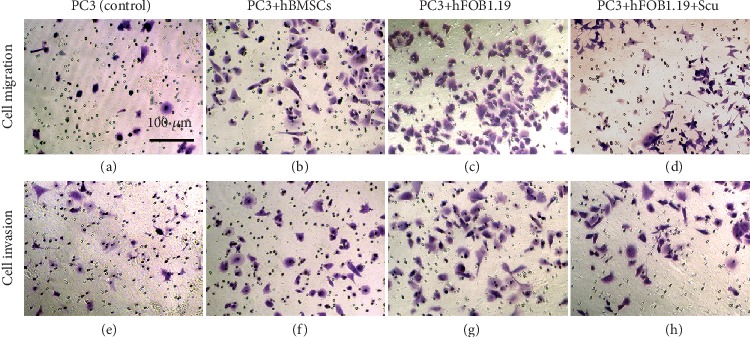
Migration and invasion of crystal violet-stained PC3 cells. The numbers of migrating and invading PC3 cells in the hBMSC and hFOB1.19 cell coculture groups are shown. The effects of scutellarin were also evaluated.

Using transwell coculture systems, we found that both hBMSC and hFOB1.19 cells significantly enhanced the migration and invasion of PC3 cells, and scutellarin inhibited these effects. The specific cell counts are shown in Figure 4, and cell growth and distributions are shown in Figure 5.

### 3.4. Expression of CD59 mRNA in PC3 Cells Is Upregulated by Coculturing Them with hBMSC or hFOB1.19 Cells

**Figure 6:**
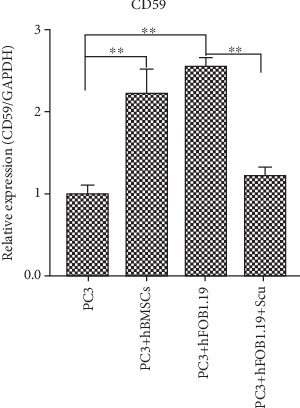
Relative expression of *CD59* mRNA in PC3 cells. The abscissa represents different treatment methods for PC3 cells. Scu: scutellarin, ^∗∗^*P* < 0.01.

CD59 protects cells from complement-induced lysis; therefore, by qRT-PCR, we calculated the effects of hBMSCs and hFOB1.19 cells on the relative expression of *CD59* mRNA by PC3 cells. We found that coculturing PC3 with either of these cell lines increased the expression of *CD59* mRNA and that this effect could be inhibited by adding the RANKL inhibitor, scutellarin, to the PC3-hFOB1.19 cocultures. The relative expression of *CD59* mRNA in each experimental group is shown in Figure 6.

### 3.5. CD59 Protein Expression Is Upregulated in PC3 Cells

**Figure 7:**
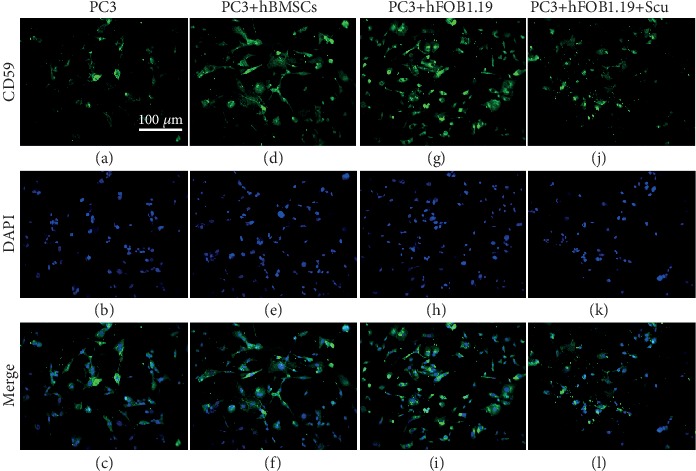
CD59 expression by immunofluorescence in PC3 cells. The numbers and fluorescence intensity of cells in the PC3 (control), PC3+hBMSC, PC3+hFOB1.19, and PC3+hFOB1.19+Scu groups are shown.

**Figure 8:**
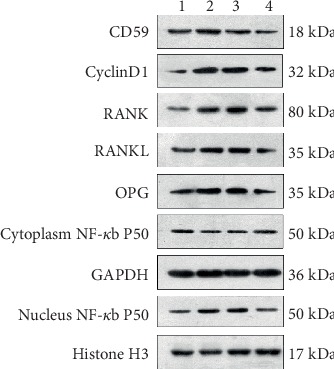
Western blot analysis. (1) PC3 control group; (2) PC3+hBMSC group; (3) PC3+hFOB1.19 group; (4) PC3+hFOB1.19+Scu group.

**Figure 9:**
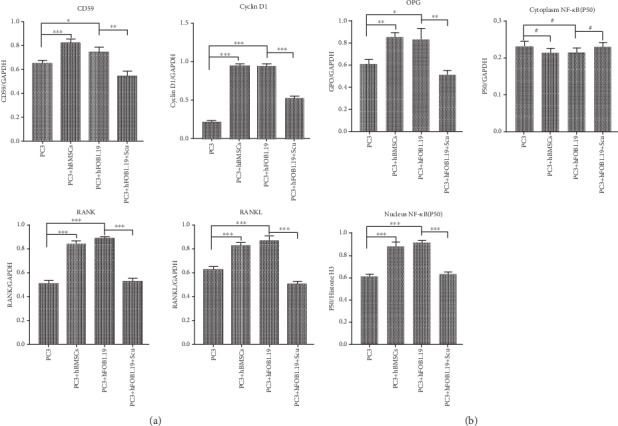
Bar graph of relative protein expression. PC3: prostate cancer cells; hBMSCs: human bone marrow mesenchymal stem cells; hFOB1.19: SV40-transfected human osteoblasts; Scu: scutellarin; GAPDH, glyceraldehyde 3-phosphate dehydrogenase, was used as an internal reference of cytoplasmic protein; Histone H3 was used as a nuclear protein loading control. ^∗^*P* < 0.05, ^∗∗^*P* < 0.01, ^∗∗∗^*P* < 0.001, and ^#^*P* > 0.05.

Immunofluorescence imaging showed that hBMSC and hFOB1.19 cells promoted the growth of PC3 cells. By both immunofluorescence (Figure 7) and western blot analysis (Figures 8 and 9), we determined that CD59 expression was significantly upregulated in PC3 cells, and these effects were blocked when the RANKL inhibitor, scutellarin, was added to the PC3-hFOB1.19 cocultures.

### 3.6. RANK Expression Is Upregulated in PC3 Cells by hBMSC and hFOB1.19 Cells

**Figure 10:**
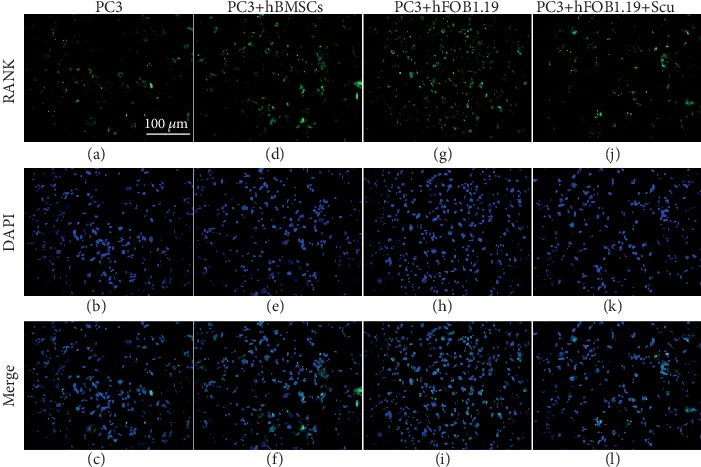
RANK expression by PC3 cells is enhanced by coculture with hBMSC and hFOB1.19 cells. The numbers and fluorescence intensity of cells in the PC3 (control), PC3+hBMSC, PC3+hFOB1.19, and PC3+hFOB1.19+Scu groups are shown.

By immunofluorescence (Figure 10) and western blotting (Figures 8 and 9), we found that PC3 cells expressed low basal levels of RANK; however, coculturing PC3 cells with hBMSCs or hFOB1.19 cells enhanced the expression of RANK by PC3 cells. Consistent with previous experiments, scutellarin significantly inhibited the effects of hFOB1.19 cells on RANK expression in PC3 cells.

### 3.7. RANKL Is Upregulated in PC3 Cells by hBMSC and hFOB1.19 Cells

**Figure 11:**
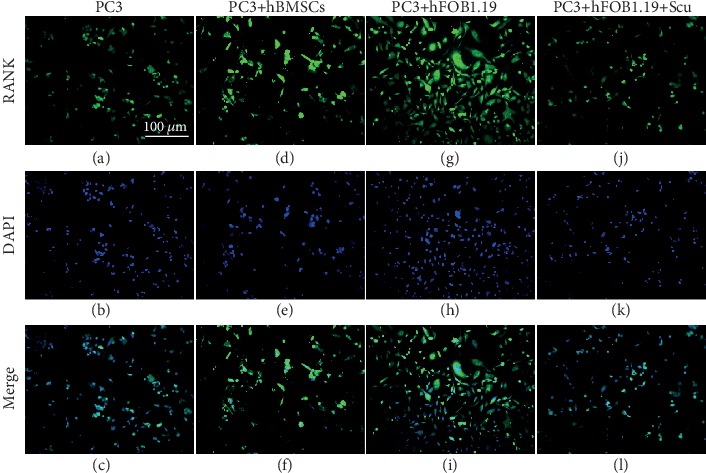
Immunofluorescence analysis of RANKL expression in PC3 cells. The numbers and fluorescence intensity of cells in the PC3 (control), PC3+hBMSC, PC3+hFOB1.19, and PC3+hFOB1.19+Scu groups are shown.

We found that RANKL was highly expressed in PC3 cells by immunofluorescence (Figure 11) and by western blotting (Figures 8 and 9). Incubating PC3 cells with hBMSCs or hFOB1.19 cells enhanced proliferation and RANKL expression in PC3 cells, while the RANKL inhibitor, scutellarin, significantly inhibited these effects in PC3-hFOB1.19 cocultures.

### 3.8. NF-*κ*B (p50) Expression by PC3 Cells Is Enhanced by hBMSC and hFOB1.19 Cells

**Figure 12:**
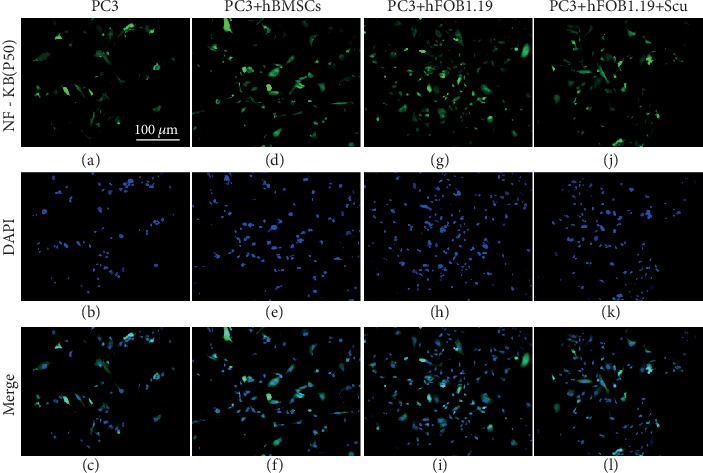
Immunofluorescence analysis of NF-*κ*B (p50) expression in PC3 cells. The numbers and fluorescence intensity of cells in the PC3 (control), PC3+hBMSC, PC3+hFOB1.19, and PC3+hFOB1.19+Scu groups are shown. Nuclear translocation of NF-*κ*B (p50) was also observed.

Using immunofluorescence imaging, we found that hBMSC and hFOB1.19 cells promoted the expression of NF-*κ*B (p50) by PC3 cells. Many of the PC3 nuclei fluoresced green, indicating that cytoplasmic NF-*κ*B had translocated to the nucleus. Consistent with previous experiments, the RANKL inhibitor, scutellarin, significantly inhibited this effect in the PC3-hFOB1.19 cocultures (Figure 12). Western blotting confirmed the enhanced expression of NF-*κ*B (p50) in the nuclei of PC3 cells; however, coculturing PC3 cells with hBMSC or hFOB1.19 cells had no significant impact on the cytoplasmic expression of NF-*κ*B (p50) (Figures 8 and 9)

## 4. Discussion

In this study, to simulate the *in vivo* interactions between prostate cancer cells and bone/bone marrow microenvironments, we cocultured PC3 prostate cancer cells with hBMSCs or hFOB1.19 cells. We found that hBMSCs and hFOB1.19 cells enhanced the proliferation, migration, and invasion of PC3 cells. Next, on the basis of this study, we will further study the effect of CD59 expression on bone metastasis of prostate cancer cells. Because PC-3 cells come from prostate cancer bone metastasis, it may have more biological characteristics of prostate cancer bone metastasis, so we chose PC-3 cells in this study.

Then, we evaluated the effects of hBMSC and hFOB1.19 cells on the cell cycle distribution of PC3 cells. We found that hBMSC and hFOB1.19 cells promoted entry of PC3 cells into the S and G_2_/M phases of the cell cycle. Cell cycle progression is regulated primarily by cyclins A, B, D, and E. D cyclins play important roles in regulating cell cycle progression and various other tumorigenic processes [11]. Mammalian cells encode three D-type cyclins (cyclin D1, cyclin D2, and cyclin D3) that are all allosteric modulators of cyclin-dependent kinases 4 and 6 (CDK4/CDK6), which coordinate the transition from G_1_ to the S phase of the cell cycle [12]. Therefore, cyclin/CDK complexes mediate the transition from quiescence (the *G*_0_ phase) to active growth and division in the G_1_, S, G_2_, and M phases [13]. Typically, in human cancers, cyclin D1 is aberrantly expressed at much higher levels than cyclin D2 or D3. Cyclin D1 overexpression leads to an imbalance in CDK activity, which accelerates cell growth (even with limited mitotic signaling), bypasses key cell cycle checkpoints, and promotes tumor growth [14, 15]. The overexpression of cyclin D1 is also associated with metastasis and poor prognosis in patients with various human cancers [16–18]. Cyclin D1 is a positive regulator of the cell cycle, acting primarily during the G_1_ phase. It promotes DNA synthesis and cell proliferation. Some studies have shown that cyclin D1 can also support the migration and invasion of cancer cells [12, 19]. In this study, we found that both hBMSCs and hFOB1.19 cells enhanced cyclin D1 expression in PC3 cells, and their effects on the proliferation of PC3 cells are consistent with a role for cyclin D1 as an enhancer of tumor cell growth. When PC3 cells were cocultured with hBMSC or hFOB1.19 cells, the percentage of PC3 cells in the G_0_/G_1_ phase decreased, whereas the percentage of cells in the S and G_2_/M phases increased, which is consistent with prior studies that demonstrated that cyclin D1 promotes cell cycle progression. We proffer that the enhanced migration and invasion of PC3 cells cocultured with hBMSC and hFOB1.19 cells may also be related to increased expression of cyclin D1.

To further characterize the mechanisms that mediate cyclin D1 expression, we analyzed changes in the RANK/RANKL/OPG signaling pathway and the expression of the p50 subunit of NF-*κ*B. RANK belongs to the tumor necrosis factor receptor superfamily and can specifically bind to its ligand, RANKL, activating the NF-*κ*B pathway. OPG is a RANKL receptor produced by osteoblasts, which competes with RANK to bind with RANKL, to inhibit the differentiation and activation of osteoclasts by RANK [20]. Compared with other members of the tumor necrosis factor family, tumor necrosis factor receptor-associated factors (TRAFs) play important roles in the initial events of the RANK-induced signal transduction pathway. TRAF1, TRAF2, TRAF3, TRAF5, and TRAF6 bind to RANK via conserved TRAF binding domains [21, 22]. Among the TRAF proteins, TRAF6 may be crucial to RANK signaling in osteoclasts because knocking out *TRAF6* leads to osteoclast differentiation and aberrant osteoclast activation [23]. TRAF proteins can transmit RANK signals to downstream targets, including NF-*κ*B. Moreover, overexpression of TRAF6 stimulates the activity of NF-*κ*B, whereas TRAF6 knockdowns suppress the activation of NF-*κ*B [21, 24, 25]. Studies have shown that NF-*κ*B directly binds to the cyclin D1 promoter to control the transcription of cyclin D1 [26].

Many studies have shown that RANK and RANKL are involved in the migration to and growth of cancer cells in the bone [27–30]. RANKL is expressed by cancer cells in prostate tumors, human bone metastases of prostate cancer, and several human prostate cancer cell lines [10, 31–33]. Prostate cancer cells release soluble RANKL and promote the formation of osteoclasts *in vitro*, although this pathway does not involve osteoblasts or bone matrix cells [34]. These data suggest that tumor-derived RANKL may play a role in mediating the metastasis of prostate cancer cells to the bone [35]. The activity of RANKL can also be regulated by OPG [36]. A study reported that serum OPG levels in patients with prostate cancer positively correlate with higher stages and grades of prostate cancer and that OPG levels in patients with bone metastases are significantly higher than in patients with localized prostate cancer or in patients with prostate cancer that has metastasized to the lymph nodes alone [37]. Importantly, our western blotting results showed that hBMSCs and hFOB1.19 cells promoted the expression of RANK and RANKL in PC3 cells. This result was also verified by immunofluorescence analysis, suggesting that there may be interactions between the bone/bone marrow microenvironment and prostate cancer cells *in vivo*. Although we found that hBMSC and hFOB1.19 cells also enhanced the expression of OPG in PC3 cells, activation or inhibition of the RANK/RANKL signaling pathway depends on the ratio of RANKL to OPG in the microenvironment [38, 39], i.e., if the increase in RANKL expression is greater than that of OPG, the RANK/RANKL signaling pathway will still be activated. Thus, an increase in OPG expression and the activation of the RANK/RANKL signaling pathway are not necessarily contradictory.

NF-*κ*B is downstream of the RANK/RANKL signaling pathway. NF-*κ*B is an important transcription factor family that includes p65 (RelA), RelB, c-Rel, p50/p105 (NF-*κ*B1), and p52/p100 (NF-*κ*B2). These transcription factors participate in various physiological and pathological processes, including inflammation, immune responses, tumor development, and tumor progression [40, 41]. Members of the NF-*κ*B family harbor a conserved Rel homologous domain, which contains five monomers, namely, RelA, RelB, cRel, p50, and p52. These monomers mediate dimerization, DNA binding, interaction with I*κ*B, and nuclear translocation. Moreover, these five monomers can form 15 potential dimer combinations. The expression of all five monomers is transcriptionally regulated; however, levels of p50 and p52 are also affected by the processing of the precursor proteins, p105 and p100, respectively. Different NF-*κ*B dimers exhibit cell type- and stimulus-specific expression, and RelA: p50, cRel: p50, and RelB: p52 are considered physiologically important dimers [40].

Immunofluorescence analysis showed that hBMSCs and hFOB1.19 cells promoted the expression of NF-*κ*B (p50) in PC3 cells. Western blotting showed that there were no changes in the expression of cytoplasmic NF-*κ*B (p50) in PC3 cells cocultured with hBMSCs and hFOB1.19 cells; however, coculturing increased the levels of nuclear NF-*κ*B (p50), indicating that NF-*κ*B was activated.

Our previous research showed that CD59 expression is higher in prostate cancer bone metastases than in primary prostate cancer lesions, which suggests that CD59 may promote prostate cancer metastasis to the bone [42]. To date, the molecular mechanisms regulating CD59 are still unclear. Recent studies have shown that *CD59* gene expression is related to NF-*κ*B activation [43, 44]. Our experiments confirmed that hBMSCs and hFOB1.19 cells increased the expression of both NF-*κ*B and CD59 by PC3 cells, which is consistent with a role for NF-*κ*B activation in the enhanced expression of CD59 in PC3 prostate cells. Upregulation of CD59 expression enables tumor cells or tumor stem cells to avoid recognition by the complement pathways [45, 46]. In addition, CD59 can also inhibit apoptosis and the neovascularization of tumors [47, 48]. In fact, CD59 is overexpressed by most tumors and very effectively protects tumor cells from complement attack [49]. Increased expression of CD59 in prostate cancer cells can facilitate evasion of the immune system, which is conducive to their growth in the bone/bone marrow microenvironments. This may be one of the possible mechanisms underlying the tendency of prostate cancer to metastasize to the bone.

To verify the relationship between the RANK/RANKL signaling pathway and the expression of cyclin D1, NF-*κ*B, and CD59, we added the RANKL inhibitor, scutellarin, to the PC3-hFOB1.19 cocultures and found that scutellarin significantly inhibited the proliferation, migration, and invasion of PC3 cells. Additionally, scutellarin reduced the proportion of cells entering the S phase and the G_2_/M phase of the cell cycle and suppressed the expression of CD59, cyclin D1, and nuclear NF-*κ*B. These findings suggest that activation of the RANK/RANKL signaling pathway promotes the expression of cyclin D1, NF-*κ*B, and CD59.

One potential mechanism underlying the tendency of prostate cancer cells to metastasize to bone is that free RANKL in the bone/bone marrow microenvironments may promote the chemotaxis of prostate cancer cells. Additionally, the migration of prostate cancer cells to the bone tissue enhances the expression of RANK, RANKL, and OPG; activates the RANK/RANKL signal pathway in an autocrine or paracrine manner; and activates the NF-*κ*B pathway, which is downstream of the RANK/RAsNKL pathway. NF-*κ*B binds to the promoter region of the gene encoding cyclin D1 to promote the transcription of cyclin D1, which decreases the ratio of cells in G_0_/G_1_ phase, increases the ratio of cells in S and G_2_/M phases, accelerates the cell cycle, and promotes cell proliferation. In addition, cyclin D1 can also enhance cell migration and invasion ^12,19^. NF-*κ*B can also stimulate the expression of CD59, and increased CD59 expression by prostate cancer cells can help them evade the immune system and promote their growth and metastasis.

## 5. Conclusion

In this study, we cocultured hBMSCs and hFOB1.19 cells with PC3 cells to simulate the physiological interactions between the bone/bone marrow microenvironment and prostate cancer cells. The effects of hBMSCs and hFOB1.19 cells on the biological behavior of prostate cancer cells were analyzed. We found that hBMSC and hFOB1.19 cells promote the proliferation, invasion, and migration of PC3 cells. They also enhanced the expression of CD59 in PC3 cells by activating the RANK/RANKL signaling pathway, which inhibited detection of PC3 cells by the immune system. However, our *in vitro* model cannot fully simulate the complex interactions between human bone marrow mesenchymal stem cells, osteoblasts, and prostate cancer cells; therefore, future studies should explore these interactions *in vivo* with a humanized mouse model of prostate cancer metastasis to the bone.

## Figures and Tables

**Table 1:** Cell cycle distribution data of PC3 cells in four different treatment groups.

Sample	Group	G_0_/G_1_ (%)	S (%)	G_2_/M (%)
PC3	PC3 (control)	74.53 ± 1.33	20.52 ± 1.29	4.95 ± 0.11
	hBMSC coculture	65.45 ± 1.69	28.39 ± 1.02	6.16 ± 0.40
	hFOB1.19 cell coculture	61.68 ± 1.64	32.27 ± 1.28	6.05 ± 0.51
	hFOB1.19 cell coculture+Scu	71.74 ± 1.32	23.17 ± 1.40	4.76 ± 0.52

## Data Availability

The figures data used to support the findings of this study are included within the article.

## Abbreviations

HRPC: Hormone-refractory prostate cancer
hBMSCs: Human bone marrow-derived mesenchymal stem cells
NF: Nuclear factor
OPG: Osteoprotegerin
RANK: Receptor activator of nuclear factor *κ*B
RANKL: RANK ligand
TRAF: Tumor necrosis factor receptor-associated factor.
